# Sex Differences in the Regulation of Interleukins in Chronic Pain: A Widely Recognized but Difficult-to-Tackle Factor

**DOI:** 10.3390/ijms26083835

**Published:** 2025-04-18

**Authors:** Jie Liu, Zheng Li, Jie Ju, Tiantian Chu, Feng Gao

**Affiliations:** 1 Department of Anesthesiology and Pain Medicine, Hubei Key Laboratory of Geriatric Anesthesia and Perioperative Brain Health, and Wuhan Clinical Research Center for Geriatric Anesthesia, Tongji Hospital, Tongji Medical College, Huazhong University of Science and Technology, Wuhan 430030, China; d202382277@hust.edu.cn (J.L.); d202282261@hust.edu.cn (Z.L.); m202276266@hust.edu.cn (J.J.); d202482507@hust.edu.cn (T.C.); 2 Department of Anesthesiology, Tongji Hospital, Tongji Medical College, Huazhong University of Science and Technology, 1095 Jiefang Ave, Wuhan 430030, China

**Keywords:** sex differences, chronic pain, interleukins, neuroinflammation

## Abstract

Chronic pain is an extremely prevalent healthcare issue that has a profound impact on individuals and society. Sex and sex hormones regulate the pain threshold differently in males and females in pain processing. However, the regulatory mechanisms of sex differences in response to painful stimuli are still unclear, which contributes to the difficulty of analgesic drug development. Interleukins mediate neuroinflammation and are involved in the development of chronic pain. Recent studies have found that sex and sex hormones are involved in the regulation of pain thresholds by interleukins. Most previous studies used male animals to study the analgesic effects of treatments due to the complexity of estrogen. This review summarizes studies that used only female animals or both sexes to examine the impact of sex on interleukin-regulated chronic pain, to provide a theoretical basis for the development of more targeted precision medicines for pain.

## 1. Introduction

Chronic pain is an extremely prevalent healthcare issue [1]. Estimates suggest that 10% of the world’s population endures chronic pain, and closer to 20–25% in individual countries and regions [2]. Chronic pain can influence the quality of life of patients and impose a substantial financial burden on the healthcare system [3,4]. Although many studies have elucidated the mechanisms underlying the development of pain [5,6,7], currently available treatments for pain are burdened by undesirable side effects. It is worth noting that most preclinical studies used male animals to explore the mechanisms of acute and persistent pain [8,9,10,11]. However, clinical studies reported that women have a higher incidence rate of chronic pain conditions, such as fibromyalgia, migraine, and osteoarthritis (OA) [12,13,14]. In addition, in one laboratory test of healthy volunteers, using various stimuli such as heat, pressure, and chemical irritants, pain thresholds and pain tolerance in women were shown to be lower than in men [15,16,17]. These indicate that women and men may have different underlying physiological mechanisms that contribute to pain. Thus, ignoring sex differences may be one of the reasons for the differences in the efficacy of pain treatment.

The interleukin (IL) family of cytokines has pleiotropic functions in inflammation and acquired immunity [18,19]. ILs can be divided into several families with more than 40 subfamily members [19]. In the nervous system, ILs are produced from many types of cells, which include neurons, glia, immune cells, and other non-neuronal cells [20]. ILs can mediate neuroimmune interaction, triggering abnormal glia activation, immune cell infiltration, and neural activation, and contributing to neuroinflammation [21]. Neuroinflammation can alter the excitability of nerve terminals and regulate pain transduction [21,22,23]. In the past several years, IL-mediated neuroinflammation has been found to participate in acute or chronic pain [22,23,24,25,26], which makes it a potential intervention candidate. Notably, the expression levels and activations of ILs are influenced by sex differences [27,28,29,30]. Furthermore, several ILs were found to exert specific effects on pain modulation in different sexes [30,31]. In this review, we will summarize the various effects of the diverse IL families in pain that are influenced by sex differences and discuss the mechanisms of these ILs in both sexes, with a special focus on females, in the hope of inspiring more individualized treatment strategies for pain.

## 2. The Influences of Sex Differences in Pain Processing and the Diversity of Estrogen Action

From a biological perspective, sexual differentiation of pain results in large part from organizational and activation effects of gonadal steroid hormones [32]. Although three common kinds of sex hormones (testosterone, estrogen, and progesterone) have been thought to be involved in nociception [32,33,34,35,36,37], their modulative mechanisms in pain processing may be different [38,39]. A previous study found that sex-related differences in spinal alpha (2)-adrenergic receptor-mediated nociception in rats were gonadal hormone-dependent: estrogen attenuated antinociceptive effects in females, whereas testosterone was required for the expression of antinociception in males [38]. This indicates the different roles of gonadal hormones in regulating pain reactivity. Notably, in another research, κ opioid receptor (KOR) antinociception was enhanced by estrogen in the spinal cord of female SD rats [39]. Selective KOR agonists could dose-dependently increase the thermal threshold in proestrus (the phase of the estrous cycle with the highest levels of circulating estradiol) females, but not in intact and ovariectomized (OVX) females or males. Furthermore, estrogen re-injection could dose-dependently enhance the effect of KOR agonist in OVX rats. These two studies reveal an interesting phenomenon: the effect of estrogen on the transmission of pain signals has a dual nature.

Estrogen receptors (ERs) include two classical receptors, ERα and ERβ [40]. A previous study showed that ERβ-selective agonists were effective in promoting allodynia induced by chemotherapy in rats, while the ERα-selective agonists had no effect on chemotherapy-induced pain [41]. This indicates that different ERs can exert different effects on pain modulation. However, the roles of these two receptors are not so absolute, and there is evidence showing that ERα and ERβ might play pro-nociceptive roles [42]. In their research, knockout of either receptor was found to significantly increase the elevated response to mechanical stimuli in female mice. The different effects of ERs are involved in different mechanisms, including protein kinase, inflammatory cytokines, and ion channels [40,43]. In addition, the different distribution of ERs may explain the mechanisms of differences. ERs are ubiquitously expressed by nociceptive system neurons [23]. However, in adult female rat ganglia, ERα expression is restricted to small sensory neurons, while ERβ is widely expressed in most sensory neurons. In the spinal cord, ERα expression appears large and numerous in the superficial dorsal horn (laminaes I–II), but ERβ is mainly expressed in the deeper laminaes (laminaes III–V, VIII, and IX) of the spinal cord [40,44,45]. Moreover, non-classical receptor GPR30 also plays a crucial role in pain modulation [40]. These results further clarify the comprehensive mechanisms of ER signaling pathways in pain processing in females.

In addition, in female rats, estrogen and progesterone regulate pain thresholds depending on their dose, the interaction between these two hormones, and the stage of pain [32,36]. Estradiol and progesterone can attenuate mechanical allodynia dose-dependently [36,46]. In inflammatory pain induced by formalin injection into the rat hind paw [36], estradiol dose-dependently exerted an analgesic effect during phase II (chronic pain) but not phase I (acute pain). Only a high dose of progesterone (500 μg) exerted an analgesic effect during phase I (acute pain) at 1% formalin. However, when co-administration of estradiol (20 μg) and progesterone, a low dose of progesterone (50 μg) could reverse the analgesic effect of estradiol, though this effect was not observed after using its high dose (500 μg). Notably, the levels and activities of estrogen and progesterone can change along with the menstrual cycle, which results in highly equivocal results about sex hormone modulation of nociception in humans and animals [32,36,47].

## 3. The Influences of Sex Differences in Interleukin Production and Reactivity in the Nervous System

Given the impracticality of treating sex hormones, we will explore other sex-associated mechanisms in pain signaling transmission. Neuroinflammation is critical for pain development [48]. ILs, produced from neurons, glia, immune cells, and other non-neuronal cells [20], play important roles in pain signaling transmission by regulating neuroinflammation [24,25,26]. Sex hormones can affect the production and signaling of interleukins by directly interacting with hormone receptors present in immune cells and neurons, which can alter gene transcription and lead to changes in cytokine expression [40,49,50,51,52]. Sex differences were found to be critical for mediating the immune system [53]. Increasingly, studies have paid attention to the roles of biological sex and sex hormones on changes in these inflammatory markers in the nervous system [54,55]. In the chronic stress model, stress exposure affected the regulation of rat brain IL-1β by the norepinephrine-β-adrenergic receptor pathway in males but not females [56]. In mice brains in which LPS induced proinflammatory cytokine response, IL-1β expression was increased in females but not males [57]. Thus, the production and activation of ILs are influenced by sex and sex hormones in different diseases, which provides important evidence that targeting sex-associated IL activations may provide a potential therapeutic strategy for sex-biased pain.

## 4. Sex Differences-Associated ILs in Chronic Pain

Chronic pain is characterized by spontaneous, ongoing, or evoked by sensory stimuli [58]. According to the international classification of diseases for chronic pain (CD-11), it can be generally categorized as chronic primary pain syndromes (such as complex regional pain syndromes (CRPS), and fibromyalgia) and chronic secondary pain syndromes [59]. The latter is linked to other diseases as the underlying cause, and it includes chronic neuropathic pain, chronic cancer-related pain, chronic musculoskeletal pain, and others. Neuropathic pain is usually caused by central nerve lesions or peripheral nerve lesions, such as peripheral nerve injury and diabetic neuropathy. Chronic cancer-related pain can be caused by the cancer itself (primary tumor or metastases) or by its treatment (surgery, chemotherapy, and radiotherapy) [58]. Chronic musculoskeletal pain is subdivided into many types according to the various underlying mechanisms, such as persistent inflammation of infectious, autoimmune, or metabolic etiology (e.g., rheumatoid arthritis), or structural changes affecting bones, joints, or muscles (e.g., symptomatic OA). Various animal models of neuropathic pain, musculoskeletal pain, cancer-related pain, and autoimmune dysfunction-induced pain have been established to explore the mechanisms underlying the development of chronic pain [26,30,60,61,62,63,64,65].

Preclinical research found that, despite various causes of different types of chronic pain, neuroinflammation was critical for most pain development [66,67]. ILs, mediators of neuroinflammation, have been well discussed in previous reviews [68,69,70], such as IL-1β [71], IL-6 [70], IL-18 [72], IL-17 [69], IL-23 [73], IL-10 [74], IL-33 [75]. However, the subjects of previous experiments have overwhelmingly been male [11]. Few studies explored the mechanisms of pain development in females or both sexes, and even identified the role of sex differences in pain processing. Thus, the hypotheses were mostly confirmed in males but not in females, indicating that the entire preclinical conclusion may be male-biased. Recently, researchers have been paying more attention to the influences of sex differences in pain development [11]. Increasing studies have focused on various roles of ILs in different sexes. For example, the IL-23/IL-17A axis could regulate female-specific mechanical pain [30]. IL-6 could mediate pain associated with posttraumatic OA in a sex-specific manner [76]. Meanwhile, studies using both sexes found that not all mechanisms contributing to pain development appeared to differ by sex [77,78]. This review focuses on the role of ILs in chronic pain females found from recent preclinical studies that only use female animals and summarizes the similarities and differences in the roles of ILs in studies that used both sexes. (Table 1).

### 4.1. IL-1β

IL-1 traditionally includes IL-1α, IL-1β, IL-1Rα, and their receptors are IL-1R1 and IL-1R2 [19]. As a proinflammatory factor, IL-1β is usually regarded as a biomarker of inflammation [104]. Estradiol and progesterone could reduce both IL-1α and IL-1β production in mononuclear leukocytes [105], indicating the effect of sex hormones in the production and activation of IL-1β.

**Figure 1:**
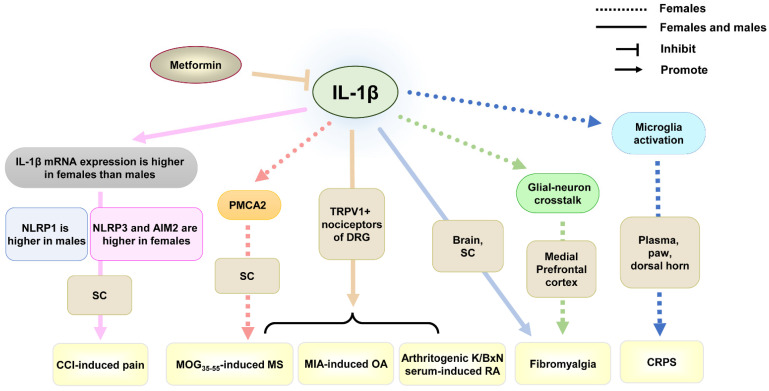
The role of IL-1β in chronic pain from studies using females only or both sexes. Abbreviations: CCI, chronic constriction injury; CRPS, complex regional pain syndromes; DRG, dorsal root ganglion; MIA, monosodium iodoacetate; MOG_35–55_, myelin oligodendrocyte glycoprotein peptide 35–55; MS, multiple sclerosis; NLRP, nod-like receptor protein; OA, osteoarthritis; PMCA2, plasma membrane calcium ATPase 2; RA, rheumatoid arthritis; SC, spinal cord; TRPV1, transient receptor potential vanilloid 1.

IL-1β was found to promote chronic pain in males, such as neuropathic pain [60,106] and inflammatory pain [61,107]. Similarly, the increased level of IL-1β in females was found in inflammatory pain and neuropathic pain [61,108] (Table 1 and Figure 1). In myelin oligodendrocyte glycoprotein peptide 35–55 (MOG_35–55_)-induced multiple sclerosis (MS), arthritogenic K/BxN serum-induced rheumatoid arthritis (RA), and monosodium iodoacetate (MIA)-induced OA models of female and male mice, IL-1β-producing myeloid cells were found to infiltrate around IL-1 receptor-expressing nociceptors in DRG. Knockout of IL-1 receptor in transient receptor potential vanilloid 1 positive (TRPV1^+^) nociceptors of DRG prevented the development of these inflammatory pains in both male and female mice [61]. Though IL-1β can participate in inflammatory pain in both males and females, the mechanisms behind it may be different. In chronic constriction injury (CCI)-induced neuropathic pain in rats, intrathecal IL-1 receptor antagonists reversed the established mechanical allodynia in both sexes. However, there were several differences in the expression level of the gene coding for IL-1β, as well as the four inflammasomes responsible for IL-1β release: nod-like receptor protein 3 (NLRP3), absent in melanoma 2 (AIM2), NLRP1, and NOD-like receptor family CARD-containing 4 protein (NLRC4) in rat spinal cord of different sexes [79]. The total mRNA level of IL-1β was higher in females than in males after CCI. NLRP3 and AIM2 proteins were more highly expressed in females, but NLRP1 expression was higher in males. These indicate that the mechanisms of IL-1β expression and activation are different in both sexes, which provides new insights into therapeutic strategies to compensate for the insufficient efficacy of IL-1 receptor antagonists in males and females. In addition, intrathecal treatment with IL-1α can dose-dependently attenuate symptoms of chronic pain by CCI or chemotherapy in male rats [109,110], which suggests the different effects between IL-1β and IL-1α. The effect of IL-1α in females needs to be further explored.

In addition, in MOG_35–55_-induced MS, an autoimmune inflammatory disorder of the nervous system, increased mechanical and thermal pain responsiveness in MS was paralleled by a significant decrease in plasma membrane calcium ATPase 2 (PMCA2) level in the spinal dorsal horn of female mice, but the PMCA2 level remained unaltered in MS mice without the increased pain [65], indicating that PMCA2 plays an important role in pain processing in MS females [80]. Though male mice showed a similar change in PMCA2 level during MS [65], female mutant PMCA2+/− mice were found to be more sensitive to evoked mechanical pain than wild-type controls of the same sex, whereas such a difference was not observed in male PMCA2+/− and PMCA2+/+ mice [80], indicating that the different mechanisms of PMCA2 in MS-associated pain in different sexes. IL-1β, tumor necrosis factor α (TNFα), and IL-6 expressions were robustly increased in the spinal dorsal horn of female mice with MS manifesting pain, whereas these cytokines showed modest increases or no change in female mice with MS in the absence of pain [65]. In vitro, only IL-1β decreased PMCA2 levels in pure spinal cord neuronal cultures, indicating that IL-1β in females can downregulate PMCA2 activation, participating in MS-associated chronic pain.

CRPS is a primary pain condition that typically affects the limb, even associated with motor dysfunction. It is usually triggered by an injury or trauma [111]. CRPS is more prevalent in females than males. In the CRPS female mouse model (Table 1), the levels of IL-1β were increased in plasma and the paw. Using IL-1 receptor antagonists, anakinra, inhibited microglia activation of the dorsal horn, not only preventing the development of CRPS, but also reversing the established CRPS [64]. This indicates that the blockade of the IL-1 receptor can alleviate CRPS in females. However, IL-1β in males was also found to participate in CRPS. The roles of IL-1β in CRPS in both sexes need to be further explored.

Fibromyalgia is a chronic primary condition characterized by widespread pain. Fibromyalgia affects approximately 3–9% of the global population and is significantly more prevalent in females than in males [112]. In the fibromyalgia model (Table 1), IL-1β in microglia of the medial prefrontal cortex of female rats was increased, and injection of IL-1β antibody significantly reduced the expression of group III secretory phospholipase A2 (sPLA2-III) in neurons, alleviating thermal hyperalgesia and mechanical allodynia in the hind paws [81], indicating that IL-1β-mediated glial-neuron crosstalk contributes to the development of fibromyalgia in females. In another study, increased IL-1β levels were found in the brain and spinal cord in both male and female mice, using metformin to ameliorate thermal hyperalgesia and mechanical allodynia. In addition, metformin significantly reduced the increased IL-1β levels in both females and males, but the IL-1β level in the brain of females, rather than males, did not reach a near-normal level [82]. These results suggest that IL-1β in both sexes can participate in the development of fibromyalgia, but the different mechanisms of IL-1β in both sexes need to be further explored.

### 4.2. IL-6

IL-6, a proinflammatory cytokine, can also participate in inflammation by binding either to the membrane-bound (classic signaling) or the soluble form (trans-signaling) of the IL-6 receptor (IL-6R) [113]. In males, upon aging, IL-6-knockout mice developed more severe spontaneous OA, compared to females [114]. In addition to the influences on IL-6 production [115,116], sex differences can regulate sex-specific responses to IL-6 [117,118,119,120]. In IL-6-knockout old-age mice, myelin basic protein level in the cerebellum was lower in females, and glial fibrillary acidic protein and lipid peroxidation levels in the hippocampus and cerebellum were increased in males, suggesting that IL-6 can exert its effects in aged females and males by regulating different mechanisms [119]. Thus, the effects of IL-6 on diseases in a sex-dependent manner make the researchers focus on sex differences for interventions and treatments in females versus males.

**Figure 2:**
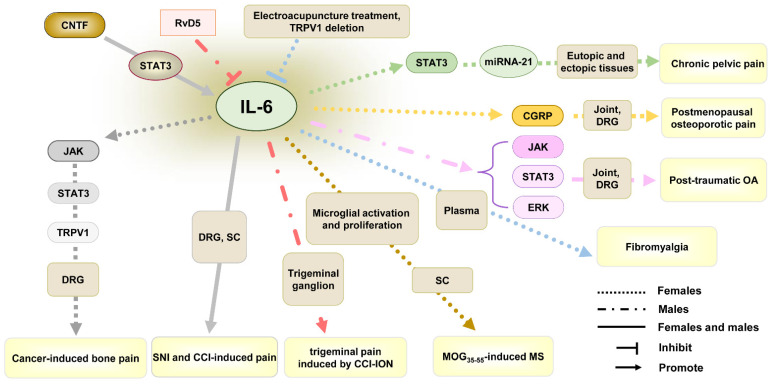
The role of IL-6 in chronic pain from studies using females only or both sexes. Abbreviations: CCI, chronic constriction injury; CCI-ION, chronic constriction injury of the infraorbital nerve; CGRP, calcitonin gene-related peptide; CNTF, ciliary neurotrophic factor; DRG, dorsal root ganglion; JAK, janus kinase; MOG_35–55_, myelin oligodendrocyte glycoprotein peptide 35–55; MS, multiple sclerosis; OA, osteoarthritis; RvD5, D-series resolvins 5; SC, spinal cord; STAT3, signal transducer and activator of transcription 3; TRPV1, transient receptor potential vanilloid 1.

IL-6/IL-6R in DRG and spinal cord were found to play important roles in chronic pain in males and females [121,122]. In cancer-induced bone pain (Table 1 and Figure 2), increased IL-6 in DRG neurons of female rats upregulated expression and activation of TRPV1 through triggering Janus kinase (JAK)/phosphatidylinositol 3-kinase (PI3K) signaling pathway, resulting in mechanical allodynia and thermal hyperalgesia [87]. This indicates that IL-6 can participate in cancer-induced bone pain in females. In neuropathic pain induced by spared sciatic nerve injury (SNI) or CCI, IL-6 participated in ciliary neurotrophic factor (CNTF)-signal transducer and activator of transcription 3 (STAT3) axis-mediated the immune cascades across the Schwann cell-neuron-microglia network in DRG and spinal cord of both males and females [83], indicating the participative roles of IL-6 in SNI or CCI-induced pain in both sexes. However, when D-series resolvins 5 (RvD5) was applied to prevent trigeminal pain induced by chronic constriction injury of the infraorbital nerve (CCI-ION), the lowest dose (3 ng) could promote the antinociceptive effect of RvD5 on heat and mechanical hyperalgesia in male rats. However, only higher doses of RvD5 (10 ng and 30 ng) could decrease chronic pain in females [89], indicating males appear to be more sensitive to RvD5, compared to females. Further research found that IL-6 level was increased in the trigeminal ganglion of male rather than female rats after CCI-ION, and RvD5 could reduce IL-6 level in melas, indicating that sex-associated IL-6 activation may play a crucial role in the efficacy difference in drug on trigeminal pain induced by CCI-ION.

In MOG_35–55_-induced MS, IL-6 expression was robustly increased in the spinal dorsal horn of female mice with MS manifesting pain [65]. Although IL-6 did not influence the PMCA2 level that can be decreased by IL-1β [65], the anti-IL-6 receptor antibody MR16-1 could decrease mechanical allodynia by inhibiting microglial activation and proliferation in the spinal cord of female MS mice [88]. This indicates the role of IL-6 in MS-associated chronic pain in females.

In inflammatory pain induced by OA, a clinical meta-analysis found that women generally reported higher pain-related symptoms [123]. When compared to men, women exhibited greater IL-6 reactivity after exposure to laboratory-evoked pain [124], which might contribute to women’s higher OA-associated pain risk. In OA induced by anterior crucial ligament transaction, IL-6 was found to regulate cartilage matrix anabolism as well as catabolism and participate in the development of OA via retinoic acid receptor-related orphan receptor-α (RORα)/IL-6/STAT3 axis in female mice [125]. However, in post-traumatic OA induced by destabilization of the medial meniscus, genetic ablation of IL-6 in female mice did not inhibit OA-associated cartilage degradation or nociceptive signaling [76]. On the contrary, IL-6 deletion in male mice reduced cartilage degradation through the attenuation of cartilage catabolism and alleviated OA-associated pain. This indicates that, though IL-6 participates in the development of OA by regulating cartilage matrix anabolism and catabolism, it seems uncertain whether IL-6 has an impact on OA-associated pain. In addition, IL-6 exerts its effect on OA not only in a sex-specific manner but also depending on the cause of OA. It should not ignore the effect of IL-6 in males with OA, though the higher level and response of IL-6 in females. Recently, many studies explored the role of JAK signaling and STAT3 signaling in IL-6-induced various types of OA in males [76,126,127]. Based on the similar regulation of IL-6 on STAT3 in females [125], there are more potential roles of IL-6 in females with other types of OA and OA-associated pain that need to be explored.

In the fibromyalgia model induced by intermittent cold stress (ICS), electroacupuncture treatment and TRPV1 deletion reversed the increase in IL-6 in female mice plasma and reduced heat and mechanical hyperalgesia [84], indicating the role of IL-6 in electroacupuncture-treated fibromyalgia. In another sex-associated pain processing, IL-6 was found to participate in postmenopausal osteoporotic pain in female mice, and using an anti-IL-6R antibody could preserve bone health and decrease osteoporotic pain by regulating calcitonin gene-related peptide (CGRP) expression in DRG [85]. In addition, IL-6 could also regulate miRNA-21 expression by STAT3 pathway and result in chronic pelvic pain induced by endometriosis in females [86]. These suggest the important role of IL-6 in chronic pain in favor of females.

### 4.3. IL-18

IL-18, a member of the IL-1 family, can bind to its specific ligand-binding chain IL-18 receptor α (IL-18Rα) and IL-18Rβ to form a heterotrimeric complex [72]. In the nervous system, IL-18 in microglia can promote neuroinflammation, leading to neurodegeneration [128]. Estradiol-17β treatment was found to improve behavioral scores of spinal cord injury by attenuating NLRP3, IL-1β, IL-18, and caspase-1 expressions [129]. IL-18 was found to be a gene linked to diseases with sex-specific prevalence [130].

An increasing number of studies have demonstrated the important role of IL-18 in the development and maintenance of chronic pain in females and males [72]. In cancer-induced bone pain, microglia in the spinal cord could maintain advanced-phase cancer pain in female rats by producing IL-18 expression to enhance synaptic transmission (Table 1) [90]. Spinal inhibition of the P2X7/p38/IL-18 pathway reduced advanced-phase bone cancer pain. In the female-specific systemic lupus erythematosus (SLE) model, activation of Gi protein-coupled receptor, GPR109A, in spinal microglia of female MRL lupus-prone mice (a well-established mouse model of human SLE) could attenuate thermal hyperalgesia via suppressing p38 activity and IL-18 production [91]. These indicate that IL-18 can participate in chronic pain induced by cancer and SLE. Most studies investigated the role of IL-18 in males [131,132]; however, its effect on chronic pain in females needs to be further explored.

### 4.4. IL-23/IL-17

IL-23, a member of the IL-12 cytokine family, is a heterodimeric cytokine composed of the IL-23p19 subunit and the IL-12/23p40 subunit [133]. IL-23 is mainly produced by activated macrophages and dendritic cells [134]. It can drive the differentiation and activation of T helper 17 (Th17) cells by binding to IL-23 receptor and IL-12 receptor β1 [133], subsequently promoting releases of IL-17A and IL-17F [134]. Estradiol can suppress IL-17A production from neutrophils and macrophages, which express estrogen receptors, resulting in psoriasis clinical phenotypes by sex-dependent differences, indicating that sex differences make the side effect risk of IL-23 inhibitors higher in females [135].

**Figure 3:**
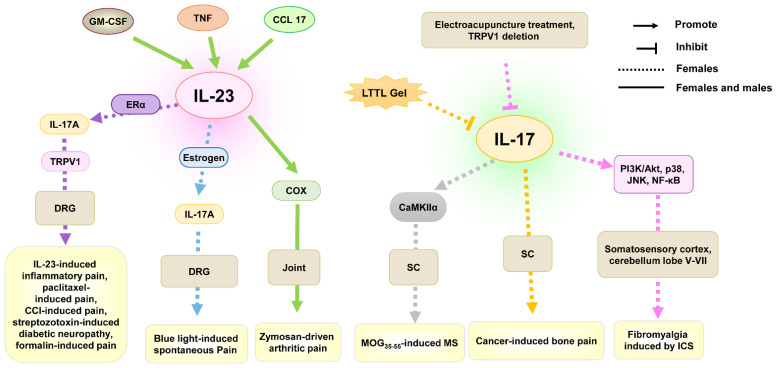
The role of IL-23/IL-17 in chronic pain from studies using females only or both sexes. Abbreviations: CCL17, C-C motif ligand 17; COX, cyclooxygenase; DRG, dorsal root ganglion; ERα, estrogen receptor α; GM-CSF, granulocyte macrophage-colony stimulating factor; ICS, intermittent cold stress; LTTL gel, gel Long-Teng-Tong-Luo; MOG_35–55_, myelin oligodendrocyte glycoprotein peptide 35–55; MS, multiple sclerosis; PI3K, phosphatidylinositol 3-kinase; SC, spinal cord; TNF, tumor necrosis factor; TRPV1, transient receptor potential vanilloid 1.

The IL-23/IL-17 axis in joints was found to play an important role in inflammatory arthritis in females and males [136]. The mechanisms of IL-23 in joints of male and female mice participating in zymosan-induced arthritic inflammatory pain were involved in granulocyte macrophage-colony stimulating factor (GM-CSF), TNF, C-C motif ligand 17 (CCL17), and cyclooxygenase (COX), which in turn have themselves been linked in this process [73,92] (Table 1 and Figure 3). Interestingly, IL-23 in the macrophages of mice DRG can induce mechanical allodynia only in females. Previous research found that intraplantar and intrathecal injections of IL-23 resulted in mechanical allodynia, but not thermal hyperalgesia or cold allodynia, in naïve female mice but not male mice in a dose-dependent manner [30]. Similarly, the IL-23/IL-23R axis was found to play an important role in chemotherapy- or CCI-induced neuropathic pain, streptozotoxin-induced diabetic neuropathy, and formalin-induced acute inflammatory pain in female mice [30]. Further research found that IL-23 promoted IL-17A release from macrophages, activating C-fiber nociceptors and TRPV1 to produce mechanical pain in females in the presence of ERα. In addition, intraplantar IL-23 could potentiate blue light-induced pain in females, and intrathecal injection of IL-23 could potentiate low-dose capsaicin-induced spontaneous pain in female but not male mice [31]. In cancer-induced bone pain, treatment with IL-17A antibody in the spinal cord of female rats could also inhibit cancer-induced mechanical allodynia and thermal hyperalgesia [93]. These indicate the importance of IL-23/IL-23R or IL-23/IL-17 axis in generating female-specific mechanical allodynia.

In fibromyalgia induced by ICS, electroacupuncture treatment and TRPV1 deletion decreased IL-17 and IL-17-related signaling pathways (PI3K/Akt, p38, JNK, NF-κB) levels in somatosensory cortex and cerebellum lobe V–VII in female mice and reduced heat and mechanical hyperalgesia [84], indicating the role of IL-17 in electroacupuncture-treated fibromyalgia in females.

In MOG_35–55_-induced MS, spinal CaMKIIα activity was enhanced in female mice, and CaMKIIα inhibitor or siRNA attenuated mechanical allodynia and thermal hyperalgesia [94]. Further research found that IL-17 induced the occurrence but not the development of mechanical allodynia and thermal hyperalgesia in the MS model, and CaMKIIα was found to participate in IL-17-mediated hyperalgesia by using a CaMKIIα inhibitor. These suggest that IL-17 can promote the occurrence of MS-associated chronic pain by improving CaMKIIα activation in females.

In addition, approximately 60–90% of individuals with psoriasis suffer from pruritus and neuropathic pain in the lesions [137,138]. The IL-23/Th17 immune axis was found to play an important role in the development of psoriasis [139]. However, no preclinical study found the role of IL-23/IL-17 in psoriasis-associated pain. Clinical trials reported that IL-23p19 neutralizing antibody, guselkumab, could reduce the physical component score that includes the bodily pain score of patients with psoriasis [140,141,142]. Psoriasis is a chronic systemic inflammatory cutaneous disease. The psychoneurological system is also influenced during the development of psoriasis [143]. IL-23/IL-17 may participate in psoriasis-associated pain through inflammation. However, the roles and mechanisms of IL23/IL-17 in psoriasis-induced pain need to be explored in females or males.

### 4.5. IL-33

IL-33, another member of the IL-1 family, is mainly produced by monocytes/macrophages [144]. It uses a receptor complex of ST2 (IL-1 receptor-like 1) and IL-1 receptor accessory protein for its receptor to participate in the inflammatory process [145]. IL-33 is regulated by sexual hormones [146]. In addition, IL-33 can also participate in menopause-induced bone loss in females [146]. Neutralizing antibody to IL-33 is effective for the treatment of endometriosis [147].

In both male and female mice, IL-33 intraplantar injection could induce mechanical hypernociception, and inhibition of TNF-α, CXCL1, or IL-1β reduced IL-33-induced mechanical allodynia [96] (Table 1). Another study explored the regulatory mechanism between IL-33 and these proinflammatory factors [95]. The IL-33/TNFα/IL-1β/IFNγ/endothelin 1-prostaglandin (PG) E2 signaling cascade was found to participate in antigen-induced cutaneous and articular hypernociception. These indicate that IL-33 in both males and females may enjoy a similar mechanism in chronic pain.

### 4.6. IL-10

IL-10 is a key anti-inflammatory cytokine, which can repress proinflammatory responses and limit unnecessary tissue inflammatory disruptions by binding to the IL-10 receptor [148,149]. IL-10 is produced by macrophages, myeloid dendritic cells, and neutrophils [150]. Sexual hormones can influence the production of IL-10 [151,152]. In the nervous system, LPS stimulation could result in different degrees of cytokine production. Astrocytes of female mice produced less IL-10, compared to those of males [153]. However, the inhibition of female hormones on IL-10 production, IL-10-deficient female mice, but not male mice, displayed increased depressive-like behavior [154], suggesting that the inhibition of female hormones on IL-10 production does not mean the unimportance role of IL-10 in females.

**Figure 4:**
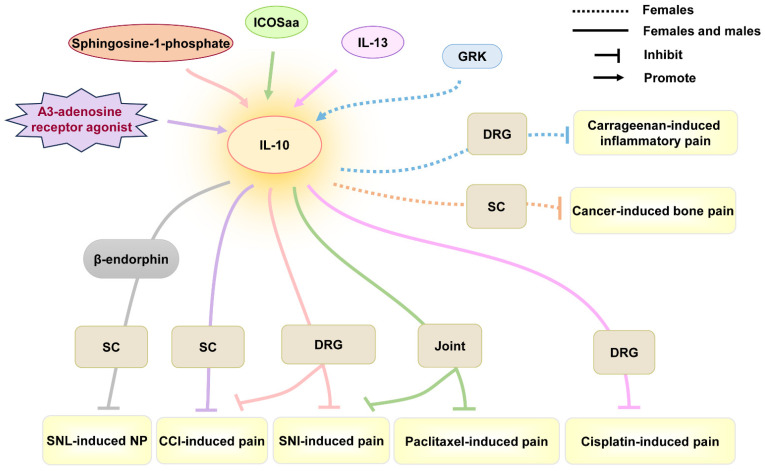
The role of IL-10 in chronic pain from studies using females only or both sexes. Abbreviations: CCI, chronic constriction injury; DRG, dorsal root ganglion; GRK, G-protein-coupled receptor kinase; ICOSaa, inducible co-stimulatory molecule agonist antibody; NP, neuropathic pain; SC, spinal cord; SNI, sciatic nerve injury; SNL, spinal nerve ligation.

In an anti-inflammatory role, IL-10 has been widely validated in chronic pain. In male and female mice, A_3_-adenosine receptor agonists promoted CD4^+^ T cells in DRG to release IL-10, reversing mechano-allodynia induced by CCI [97]. Sphingosine-1-phosphate antagonists could promote IL-10 production of astrocytes in the spinal cord, attenuating and reversing neuropathic pain induced by CCI or SNI in both female and male mice [98]. Similarly, IL-10 reversed spinal abnormal synaptic plasticity of female and male rats through increasing β-endorphin expression in microglia, contributing to the inhibition of hypersensitivity activity in neuropathic pain induced by spinal nerve ligation (SNL) [99]. In chemotherapy-induced pain, IL-13 produced by CD8^+^ T cells promoted IL-10 release in macrophages of DRG, ameliorating cisplatin-induced mechanical allodynia in both male and female mice [62]. These suggest the anti-inflammatory effect of IL-10 in both sexes (Table 1 and Figure 4). However, the efficiency of IL-10 may be different between the two sexes. Inducible co-stimulatory molecule agonist antibody (ICOSaa) alleviated mechanical hypersensitivity in paclitaxel-induced pain by recruiting IL-10-producing T cells, but ICOSaa applied in female mice showed a more effective pharmacological effect [100]. In addition, intrathecal IL-10 could effectively reduce cancer-induced bone pain behavior in female rats, in a dose-dependent manner [63]. IL-10 produced by peripheral monocytes/macrophages in DRG promoted the resolution of carrageenan-induced mechanical allodynia and thermal hyperalgesia in female mice [101]. Thus, IL-10 can alleviate the development of chronic pain, though slight differences caused by sex differences.

IL-10 was also found to play an anti-inflammation role in MS by decreasing microglial activation, T-cell proliferation, peripheral immune cell infiltration, or IFN-γ secretion [155]. Plasmid construct coding for rat interleukin-10 (pDNA-IL-10F129S) could reduce mechanical allodynia in MS induced by MOG_35–55_ in male rats. However, the role of IL-10 in MS-associated pain in females needs to be explored. In addition, IL-10 was found to be increased during pharmacological therapy that inhibited pain-like behavior in osteoarthritis in female or male rats [156,157]. Thus, the role of IL-10 in autoimmune disease-associated chronic pain needs to be further explored.

### 4.7. IL-35

IL-35 is another anti-inflammatory effector cytokine, which plays an immunosuppressive role. IL-35 is released by regulatory T cells, regulatory B cells, and tolerogenic dendritic cells [102]. IL-35 treatment could regulate microglia activation, reducing spinal neuroinflammation and alleviating diabetic neuropathic pain in male rats [158]. In CCI-induced neuropathic pain, intrathecal recombinant IL-35 treatment alleviated mechanical pain in male mice but not female mice [102]. Further research found that IL-35 could inhibit microglia activation in the spinal cord of males but not females, indicating that IL-35 can participate in CCI-induced neuropathic pain in males rather than females (Table 1). However, in MOG_35–55_-induced MS, IL-35 reduced mechanical allodynia and spontaneous pain and increased myelination of the nociceptive pathway in female mice [103]. Further research found that IL-35 upregulated IL-10 expression and reduced monocyte infiltration in the spinal cord, indicating that IL-35 can attenuate MS-associated pain in females by decreasing neuroinflammation and increasing myelination.

## 5. Therapeutic Strategy of IL Family

Some strategies have been applied to reverse the effects of the IL family, such as naturally occurring proteins, neutralizing antibodies, or recombinant receptor antagonists [159,160]. These treatments for the IL family in clinical trials showed outstanding therapeutic efficacy [159,160,161,162,163,164]. However, whether these treatments can exert their effects on chronic pain needs to be further explored. Currently, IL-6R inhibitors, such as olokizumab and sirukumab, have been used in efficacy studies in randomized phase III safety trials and demonstrated a significant superiority in primary efficacy outcome in rheumatoid arthritis, compared to the placebo group [165]. Clinical trials reported that IL-23p19 neutralizing antibody, guselkumab, could reduce the physical component score, including the bodily pain score, of patients with psoriasis [140,141,142]. Notably, drugs that target the IL-23/IL-17 pathway showed excellent efficacy for skin disease, efficacy for inflammatory bowel disease, and peripheral arthropathy associated with spondyloarthropathy [166]. However, the female sex increased the risk of drug discontinuation [135,167]. This makes the researchers pay more attention to the influence of sex in drug efficacy and side effects. In addition, preclinical studies showed that, in addition to ILs/IL-Rs itself invention, the upstream of the IL family should be taken into account for pain treatment [90,98,125,168]. However, females and males showed different IL reactivities in pain processing [123,124,169,170] and this review summarized the different effects of ILs in pain transmission in both sexes, especially in females (Table 1). Researchers should focus on sex differences during developing interventions and treatments for chronic pain.

## 6. The Change in Pain Threshold in Klinefelter Syndrome

Klinefelter syndrome (KS) is the most frequently observed chromosomal disorder in males [157]. The most common form of KS is the regular type (47, XXY), which accounts for 80% of all cases. The other common forms of KS are 47, XX and, der(Y), 47, X, der(X), Y,48, XXXY, 48, XXYY, 49, XXXXY, 47, XXY/46, and XY mosaicism. KS is generally characterized by tall stature, small testes, gynecomastia, and infertility. Serum analysis showed lower testosterone, and higher serum follicle-stimulating hormone and luteinizing hormone levels, which are accompanied by impaired spermatogenesis [171]. Sex hormones affect immune cells and responses, resulting in differences in immune cell compositions and functions in different sexes. Females generally mount stronger immune responses than males [172], and females are much more susceptible to autoimmune diseases. Thus, the additional X-chromosomes make males with KS to develop autoimmune diseases, such as systemic lupus erythematosus, as frequently as women [172,173,174,175]. However, the effect of this type of X chromosome aneuploidies on pain threshold is unclear. Recent clinical research found that, at 30, 60, and 90th min after surgery, visual analog scale pain scores of males with KS were higher than those of males with a normal karyotype [176], indicating that males with KS are more sensitive to painful stimuli. However, the role of KS in chronic pain needs to be further explored.

## 7. Conclusions

Neuroinflammation plays an important role in pain plasticity, and the IL family plays a crucial regulatory role in this process. Recently, the impact of sex differences on pain processing has been increasingly recognized. Sex and sex hormones can regulate IL production and reactivity. This review summarizes the impacts of sex differences on IL family regulation in chronic pain. The roles and mechanisms of some ILs in chronic pain are different in males and females. In addition, the different causes of chronic pain can influence the effects of ILs in the same sex. These provide insights and considerations for the development of precise therapeutic drugs for chronic pain.

## Figures and Tables

**Table 1:** IL-mediated chronic pain in studies using only female animals or both sexes.

Sex	Interleukin	Mechanism	Effect on Pain	Type of Pain Model	Type of Pain-Like Behavior	Location	Expression Patterns	Reference
Female and male	IL-1β	Knockout of IL-1 receptor in TRPV1^+^ nociceptors of DRG prevents the development of inflammatory pain.	Promote	MOG_35–55_-induced MS, arthritogenic K/BxN serum-induced RA, and MIA-induced OA	Mechanical allodynia	TRPV1^+^ nociceptors of DRG	Up-regulation	[61]
Female and male	IL-1β	Total mRNA expression of IL-1β is higher in females than in males after CCI. NLRP3 and AIM2 are more highly expressed in females, but NLRP1 expression is higher in males.	Promote	CCI-induced pain	Mechanical allodynia	SC	Up-regulation	[79]
Female, but not male	IL-1β	IL-1β increases mechanical and thermal pain responsiveness by decreasing PMCA2 levels.	Promote	MOG_35–55_-induced MS	Mechanical and thermal hyperalgesia	SC	Up-regulation	[65,80]
Female	IL-1β	IL-1 receptor antagonists prevent the development of CRPS and reverse the established CRPS by inhibiting microglia activation of dorsal horn.	Promote	CRPS	Mechanical allodynia	plasma, paw, dorsal horn	Up-regulation	[64]
Female	IL-1β	IL-1β-mediated glial-neuron crosstalk contributes to the development of fibromyalgia.	Promote	Fibromyalgia	Thermal hyperalgesia, mechanical allodynia	medial prefrontal cortex	Up-regulation	[81]
Female and male	IL-1β	Metformin ameliorates fibromyalgia by reducing the increased IL-1β levels in males, but partially decreasing IL-1β levels in the brain of females.	Promote	Fibromyalgia	Thermal hyperalgesia, mechanical allodynia	Brain, SC	Up-regulation	[82]
Female and male	IL-6	CNTF-STAT3-IL-6 axis.	Promote	SNI or CCI-induced pain	Mechanical allodynia, thermal hyperalgesia	DRG, SC	Up-regulation	[83]
Male, but not female	IL-6	IL-6/JAK signaling is a critical mediator of IL-6-induced cartilage catabolism and pain signaling in nociceptive neurons; IL-6/STAT3 signaling is a potent driver of cartilage catabolism; IL-6/ERK signaling is essential for IL-6-induced neurite outgrowth and pain signaling in DRG neurons.	Promote	Post-traumatic OA induced by destabilization of the medial meniscus	Mechanical allodynia	Joint, DRG	Up-regulation	[76]
Female	IL-6	Electroacupuncture treatment and TRPV1 deletion reduce chronic pain by reversing the increase in IL-6.	Promote	fibromyalgia induced by ICS	Mechanical allodynia, thermal hyperalgesia	plasma	Up-regulation	[84]
Female	IL-6	IL-6 induces postmenopausal osteoporotic pain by regulating calcitonin gene-related peptide (CGRP) expression.	Promote	Postmenopausal osteoporotic pain	Mechanical allodynia	Joint, DRG	Up-regulation	[85]
Female	IL-6	IL-6 regulates miRNA-21 expression by STAT3 pathway and results in chronic pelvic pain induced by endometriosis	Promote	Chronic pelvic pain	/	Eutopic and ectopic tissues	Up-regulation	[86]
Female	IL-6	IL-6 upregulates TRPV1 expression and function through JAK/PI3K signaling pathway.	Promote	Cancer-induced bone pain	Mechanical allodynia, thermal hyperalgesia	DRG	Up-regulation	[87]
Female	IL-6	Anti-IL-6 receptor antibody decreases mechanical allodynia by inhibiting microglial activation and proliferation.	Promote	MOG_35–55_-induced MS	Mechanical allodynia	SC	Up-regulation	[88]
Male, but not female	IL-6	RvD5 can inhibit trigeminal pain by reducing level of IL-6.	Promote	Trigeminal pain induced by CCI-ION	Mechanical allodynia, thermal hyperalgesia	Trigeminal ganglion	Up-regulation	[89]
Female	IL-18	Microglia can maintain advanced-phase cancer pain by producing the proinflammatory cytokine IL-18 to enhance synaptic transmission.	Promote	Cancer-induced bone pain	Mechanical allodynia, thermal hyperalgesia	Microglia in SC	Up-regulation	[90]
Female	IL-18	Gi protein-coupled receptor (GPR109A) attenuates thermal hyperalgesia via suppressing p38 MAPK activity and production of IL-18.	Promote	SLE	Thermal hyperalgesia	Microglia in SC	Up-regulation	[91]
Female and male	IL-23	IL-23 promotes arthritic inflammatory pain induced by GM-CSF, TNF, or CCL17 via COX.	Promote	Zymosan-driven arthritic pain	Pain-like behavior (incapacitance meter)	Joint	Up-regulation	[73,92]
Female, but not male	IL-23/IL-17A	Under expression of ERα, IL-23 requires IL-17A release from macrophages to evoke mechanical pain through TRPV1 nociceptor.	Promote	IL-23-induced pain, chemotherapy (paclitaxel)-induced pain, CCI-induced pain, streptozotoxin-induced diabetic neuropathy, formalin-induced pain	Mechanical allodynia, but not thermal hyperalgesia, or cold allodynia	IL-23 in macrophages of DRG, IL-17A in C-fiber nociceptors of DRG	Up-regulation	[30]
Female, but not male	IL-23	Estrogen and IL-23 co-application increases IL-17A release in THP-1 human macrophages and promotes C-fiber-mediated spontaneous pain.	Promote	Blue light-induced spontaneous Pain	Mechanical allodynia	C-fiber nociceptors of DRG	Up-regulation	[31]
Female	IL-17A	Chinese medicated gel Long-Teng-Tong-Luo inhibits bone cancer pain by decreasing transient receptor potential channel expression in DRG and spinal astrocyte IL-17A.	Promote	Cancer-induced bone pain	Mechanical allodynia, thermal hyperalgesia	SC	Up-regulation	[93]
Female	IL-17	Electroacupuncture treatment and TRPV1 deletion reduce heat and mechanical hyperalgesia by decreasing IL-17 and IL-17-related signaling pathways (PI3K/Akt, p38, JNK, NF-κB) levels.	Promote	Fibromyalgia induced by ICS	Mechanical allodynia, thermal hyperalgesia	Somatosensory cortex, cerebellum lobe V-VII	Up-regulation	[84]
Female	IL-17	IL-17 promotes the occurrence of MS-associated chronic pain by improving CaMKIIα activation.	Promote	MOG_35–55_-induced MS	Mechanical allodynia, thermal hyperalgesia	SC	Up-regulation	[94]
Female and male	IL-33	IL-33-TNFα-IL-1β-IFNγ-endothelin 1-prostaglandin (PG) E2 signaling cascade participates in antigen-induced cutaneous and articular hypernociception.	Promote	Antigen-induced pain	Mechanical allodynia	Skin of paw	Up-regulation	[95,96]
Female and male	IL-10	A_3_-adenosine receptor agonist reverses mechanical allodynia by promoting the IL-10 release of CD4^+^ T cells in DRG.	Reverse	CCI-induced pain	Mechanical allodynia	CD4^+^ T cells in DRG	/	[97]
Female and male	IL-10	Sphingosine-1-phosphate antagonists attenuate and reverse neuropathic pain by promoting IL-10 production in astrocytes of spinal cord.	Alleviate, reverse	SNI- and CCI-induced pain	Mechanical allodynia, thermal anti-nociception	Astrocytes in SC	/	[98]
Female and male	IL-10	IL-10 inhibits spinal abnormal synaptic plasticity through β-endorphin expression in microglia.	Alleviate	SNL-induced pain	Mechanical allodynia, thermal hyperalgesia	SC	/	[99]
Female and male	IL-10	Inducible co-stimulatory molecule agonist antibody (ICOSaa) shows a more rapid resolution of mechanical hypersensitivity in females by recruiting T cells and driving IL-10 production.	Alleviate	Chemotherapy (paclitaxel)-induced pain, SNI-induced pain	Mechanical allodynia	T cell in DRG	/	[100]
Female and male	IL-10	IL-13 produced by CD8^+^ T cells promotes IL-10 release in macrophages of DRG, ameliorating cisplatin-induced mechanical allodynia.	Alleviate	Chemotherapy (cisplatin)-induced pain	Mechanical allodynia	Macrophages of DRG	Up-regulation	[62]
Female	IL-10	Intrathecal IL-10 can effectively reduce cancer-induced bone pain behavior.	Alleviate	Cancer-induced bone pain	Mechanical allodynia	SC	/	[63]
Female	IL-10	IL-10 from GRK^+^ macrophages promotes resolution of carrageenan-induced mechanical allodynia and thermal hyperalgesia.	Alleviate	Carrageenan-induced pain	Mechanical allodynia, thermal hyperalgesia	Peripheral monocytes/macrophages in DRG	Up-regulation	[101]
Male	IL-35	Intrathecal recombinant IL-35 treatment alleviates mechanical pain by inhibiting microglia activation.	Alleviate	CCI-induced pain	Mechanical allodynia	SC	/	[102]
Female	IL-35	IL-35 reduces mechanical allodynia and spontaneous pain by increasing myelination, upregulating IL-10 expression, and reducing monocyte infiltration.	Alleviate	MOG_35–55_-induced MS	Mechanical allodynia, spontaneous pain	SC	Down-regulation	[103]

AIM2, absent in melanoma 2; CCI, chronic constriction injury; CCI-ION, chronic constriction injury of the infraorbital nerve; CCL17, C-C motif ligand 17; DRG, dorsal root ganglion; ERα, estrogen receptor subunit α; GM-CSF, granulocyte macrophage-colony stimulating factor; ICS, intermittent cold stress; JAK, Janus kinase; MIA, monosodium iodoacetate; MOG_35–55_, myelin oligodendrocyte glycoprotein peptide 35–55; MS, multiple sclerosis; NLRP1, nod-like receptor protein 1; NLRP3, nod-like receptor protein 3; OA, osteoarthritis; PI3K, phosphatidylinositol 3-kinase; RA, rheumatoid arthritis; RvD5, D-series resolvins 5; SC, spinal cord; SLE, systemic lupus erythematosus; SNL, spinal nerve ligation; TNF, tumor necrosis factor; TRPV1, transient receptor potential vanilloid channel type 1.

## Data Availability

Not applicable.
